# Declines in Sugar-Sweetened Beverage Consumption Among Children in Los Angeles County, 2007 and 2011

**DOI:** 10.5888/pcd10.130049

**Published:** 2013-08-08

**Authors:** Paul A. Simon, Amy S. Lightstone, Steve Baldwin, Tony Kuo, Margaret Shih, Jonathan E. Fielding

**Affiliations:** Author Affiliations: Amy S. Lightstone, Steve Baldwin, Tony Kuo, Margaret Shih, Jonathan E. Fielding, Los Angeles County Department of Public Health, Los Angeles, California.

## Abstract

This study assessed changes in consumption of sugar-sweetened beverages (SSBs) among children (aged ≤17 years) in Los Angeles County. We analyzed children’s data from the 2007 (n = 5,595) and 2011 (n = 5,934) Los Angeles County Health Survey. The percentage of children who consumed 1 or more SSB per day decreased from 43.3% in 2007 to 38.3% in 2011 (*P* < .001); this decrease was seen across most sociodemographic subgroups. Despite measurable progress in reducing SSB consumption among children in Los Angeles County, consumption remains high, highlighting the need for additional policy and programmatic interventions.

## Objective

Given recent evidence of the link between consumption of sugar-sweetened beverages (SSBs) and obesity (1), reducing SSB consumption has become a focus of childhood obesity prevention efforts. For example, California legislation prohibiting the sale of most SSBs on school campuses became effective in 2007 (2). In Los Angeles County, this policy was accompanied by intensive education on SSBs in schools, preschools, childcare sites, and other community settings, and in the Special Supplemental Nutrition Program for Women, Infants, and Children (WIC), which serves more than 50% of children younger than 5 years in the county (Shannon Whaley, PhD, Director of Research and Evaluation, PHFE WIC, Los Angeles County, March 20, 2013). To assess the collective effect of these and other efforts, we examined data on SSB consumption among children in the county from the 2007 and 2011 Los Angeles County Health Survey (LACHS).

## Methods

The LACHS is a periodically administered, random-digit–dial telephone survey of the noninstitutionalized county population and includes both adult and child (aged ≤17 years) components (3). We analyzed data from the child components of the 2 most recent surveys, conducted in 2007 and 2011 with 2 independent samples (n = 5,595 in 2007 and 5,934 in 2011). The study was approved by the Los Angeles County Department of Public Health’s institutional review board.

For each survey, 1 child was randomly selected from each sampled household, and data were reported by the parent or other adult in the household who was most knowledgeable about the child’s health and well-being (henceforth referred to as “primary caretaker”). The surveys included identically worded questions on sociodemographic characteristics and SSB consumption (“On an average day, about how many sodas or other sweetened drinks such as Gatorade, Red Bull, or Sunny Delight does [child] drink? Do not include diet sodas or sugar-free drinks. Please count a 12-ounce can, bottle, or glass as 1 drink.”). The SSB consumption question was adapted from a question used in the 2005 New York City Community Health Survey. Interviews were conducted in English, Spanish, Cantonese, Mandarin, Vietnamese, and Korean. Among all eligible households contacted, interviews were completed for 40% in 2007 and 64% in 2011.

There were 2 methodologic differences between the 2 surveys. First, only the 2011 survey included cellular telephones, which accounted for 4% of the interviews. Second, a more sophisticated raking procedure was adopted in weighting the survey in 2011 (4). These changes were made to maintain survey representativeness and validity and are similar to those made to the Behavioral Risk Factor Surveillance System in 2011 (5). Further details regarding the survey design and changes in weighting methods are reported elsewhere (3,4).

The percentage of children who consumed 1 or more SSB per day was calculated for the total study group in each survey and by sex, age group, child’s race/ethnicity, annual household income relative to the federal poverty level (FPL), and primary caretaker’s level of education. Among Latinos, results were further stratified by the language of interview (English or Spanish). Differences in percentages were assessed for significance by using the χ^2^ test, and 95% confidence intervals were calculated for each point estimate. All analyses were conducted using SAS (version 9.2, SAS Institute Inc, Cary, North Carolina) using child sample weights; significance was set using an α of .05.

## Results

In both 2007 and 2011, the percentage of children who consumed 1 or more SSB per day was highest among children aged 12 to 17 years and lowest among those aged 5 years or younger (Table). The percentage of children who consumed 1 or more SSB per day was inversely related to household income and primary caretaker’s education level. The percentage was higher among Latino and African American children than among white and Asian/Pacific Islander children.

**Table:** Sugar-Sweetened Beverage (SSB) Consumption Among Children in Los Angeles County, 2007 and 2011

Characteristics	2007		2011		% Change	*P* Value
	n^a^	% Who Consumed ≥1 SSB Per Day (95% CI)	n^a^	% Who Consumed ≥1 SSB Per Day (95% CI)		
**Sex**						
Male	2,862	46.4 (44.2–48.5)	3,048	41.4 (38.4–44.4)	−10.8	<.001
Female	2,733	40.0 (37.8–42.2)	2,886	35.1 (32.0–38.1)	−12.3	<.001
**Age, y**						
0-5	1,736	28.6 (25.9–31.2)	1,689	23.9 (20.2–27.6)	−16.4	.001
6-11	1,810	45.2 (42.5–47.9)	1,830	39.6 (36.0–43.3)	−12.4	<.001
12–17	2,049	55.7 (53.1–58.3)	2,415	50.4 (46.9–53.8)	−9.5	<.001
**Race/ethnicity**						
Latino	3,374	49.2 (47.2–51.2)	3,212	42.3 (39.5–45.2)	−14.0	<.001
White	1,217	28.8 (25.9–31.7)	1,490	26.3 (22.7–29.9)	−8.7	.18
African American	371	53.7 (47.2–60.1)	572	48.5 (40.7–56.3)	−9.7	.10
Asian/Pacific Islander	541	25.7 (21.5–29.9)	640	28.2 (22.9–33.6)	9.7	.33
**Language of interview among Latinos**						
Spanish	2,172	54.3 (51.9–56.7)	1,652	45.5 (41.6–49.4)	−16.2	<.001
English	1,040	40.8 (37.3–44.3)	1,289	40.5 (35.8–45.2)	−0.7	.91
**Annual household income, % of FPL**						
<300	3,955	48.6 (46.8–50.5)	3,463	43.0 (40.2–45.7)	−11.5	<.001
≥300	1,640	29.0 (26.4–31.5)	2,471	28.2 (25.2–31.1)	−2.8	.61
**Primary caretaker’s education level**						
Less than high school	1,705	57.0 (54.3–59.8)	1,307	49.1 (44.5–53.6)	−13.9	<.001
High school graduate	1,017	43.1 (39.4–46.8)	1,034	46.2 (41.1–51.4)	7.2	.14
Some college or trade school	1,192	40.7 (37.3–44.1)	1,334	40.4 (35.6–45.2)	−0.7	.89
College or postgraduate	1,607	29.1 (26.5–31.8)	2,215	23.2 (20.7–25.7)	−20.3	<.001
**Total**	5,595	43.3 (41.7–44.8)	5,934	38.3 (36.2–40.4)	−11.5	<.001

Abbreviations: CI, confidence interval; FPL, federal poverty level.

a Values may not sum to totals due to missing data.

Among all children, the percentage who consumed 1 or more SSB per day decreased from 43.3% in 2007 to 38.3% in 2011 (*P* < .001). Similar declines were seen across sex and age groups and among Latino children in households interviewed in Spanish (Table). Declines were also seen among children whose primary caretaker had less than a high school education or a college or postgraduate degree. A decrease was also observed among children from households whose incomes were below 300% of the FPL (*P* < .001) but not among those from households whose incomes were at or above 300% of the FPL (*P* = .61).

## Discussion

Our findings suggest measurable progress in reducing SSB consumption among children in Los Angeles County. In particular, the decline in consumption among Latino children for whom interviews were conducted in Spanish and the decrease in consumption among children living in low-income households suggest that community education efforts in the county may be effective. For example, a public education campaign, Rethink Your Drink, launched in 2009, targeted low-income communities and included a prominent Spanish language component. In addition, the WIC programs in the county have provided intensive bilingual education on SSBs since 2007. These efforts were further buttressed by education and environmental strategies to reduce SSB consumption in the county that were supported by the federal Communities Putting Prevention to Work initiative, which started in 2010.

Despite this modest progress, the percentage of children who daily consume SSBs remains high and is consistent with several national studies (6,7), highlighting the need for more effective interventions. Community education accompanied by environmental strategies to reduce access to and demand for SSBs, including interventions in school and childcare settings, are likely to have the greatest effect, as are efforts to counter the influence of beverage industry marketing practices (8).

This study had several limitations. First, the findings are based on parent reports and, therefore, may be subject to inaccurate reporting of consumption and social desirability bias. However, the trends are consistent with an earlier study in California (9) and with reported declines in soft drink sales (10). Second, the low response rates may have introduced bias. However, the demographic characteristics of the 2 samples were similar to the county population. In addition, sample weights were adjusted to account for differential probability of selection, nonresponse, noncoverage, and type of telephone service. Third, the study was ecologic and lacked a control group, limiting its ability to confirm a causal relationship between the decline in SSB consumption and temporally associated public health interventions.

Although the study was limited to a single county, its population is the largest and one of the most diverse of any local jurisdiction in the nation. In addition, it is a setting where substantial education and policy efforts have been undertaken to reduce SSB consumption in children. The findings suggest that, although these efforts have had a measurable effect, additional policy and programmatic interventions are needed in the interest of improved child health and development.
